# Alternative substrate kinetics of SARS-CoV-2 Nsp15 endonuclease reveals a specificity landscape dominated by RNA structure

**DOI:** 10.1093/nar/gkae939

**Published:** 2024-10-30

**Authors:** Nidhi Kalia, Kimberly C Snell, Michael E Harris

**Affiliations:** Department of Chemistry, University of Florida, Gainesville, FL, 32611, USA; Department of Chemistry, University of Florida, Gainesville, FL, 32611, USA; Department of Chemistry, University of Florida, Gainesville, FL, 32611, USA

## Abstract

Coronavirus endoribonuclease Nsp15 contributes to the evasion of host innate immunity by suppressing levels of viral dsRNA. Nsp15 cleaves both ssRNA and dsRNA *in vitro* with a strong preference for unpaired or bulged U residues, and its activity is stimulated by divalent ions. Here, we systematically quantified effects of RNA sequence and structure context that define its specificity. The results show that sequence preference for U↓A/G, observed previously, contributes only *ca*. 2-fold to *k*_cat_/*K*_m_. In contrast, dsRNA structure flanking a bulged U residue increases *k*_cat_/*K*_m_ by an order of magnitude compared to ssRNA while base pairing in dsRNA essentially blocks cleavage. Despite enormous differences in multiple turnover kinetics, the effect of RNA structure on the cleavage step is minimal. Surprisingly, although divalent ion activation of Nsp15 is widely considered to be important for its biological function, the effect on *k*_cat_/*K*_m_ is only ∼2-fold and independent of RNA structure. These results reveal a specificity landscape dominated by RNA structure and provide a quantitative framework for identifying interactions that underlie specificity, determining mechanisms of inhibition and resistance and defining targets important for coronavirus biology.

## Graphical Abstract

**Graphical Abstract:**
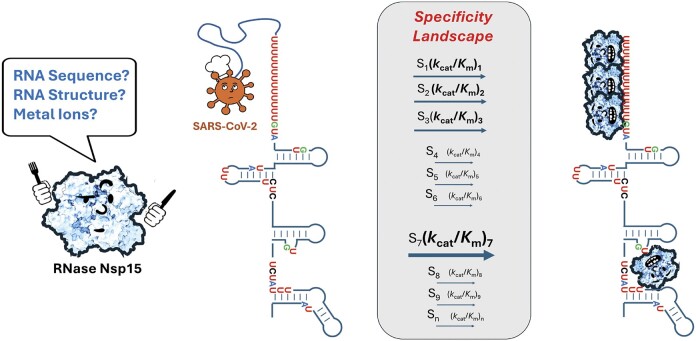

## Introduction

Non-structural protein 15 (Nsp15) is a conserved uridine-specific RNA endonuclease encoded by coronaviruses that display broad specificity that is fundamental to its function in viral immune evasion (1–3). During coronavirus infection, Nsp15 contributes to the evasion of the host immune response in part by preventing the accumulation of dsRNA intermediates (1,4–6) but may contribute through multiple mechanisms (7,8), including roles that are independent of its catalytic activity (9). Moreover, the full range of its RNA substrates in the transcriptomes of infected cells is not known. Nonetheless, the available functional data show that the RNase activity of Nsp15 is key for viral infection and therefore essential to understanding coronavirus biology and a valuable target for antiviral development. Understanding the biological role of Nsp15 and inhibitor discovery can be advanced by defining principles that quantitatively describe its RNA substrate specificity and the reaction kinetics of model RNA substrates. Deconvoluting principles of Nsp15 specificity requires quantitative measurements of rate constants aimed at isolating the contributions of RNA sequence and structure that can direct the modeling of alternative target site selection (10,11). Such knowledge is essential for understanding how it selects substrate cleavage sites that are important for its role in coronavirus biology and as a context for establishing structure–function relationships that underlie specificity.

The Nsp15 from SARS-CoV-2 is a 39 kDa monomer that assembles into a hexamer, formed by a dimer of trimers (2). Mutations that block assembly generally block ribonuclease activity (12–18), demonstrating that oligomerization is required for activity; however, low levels of monomer activity have been reported (18). Early studies showed that unpaired U residues are preferentially cleaved by Nsp15 and provided initial evidence that RNA structure could impact cleavage site selection (14,19). Structures of Nsp15 bound to uridine nucleotides revealed the molecular basis for U nucleobase specificity and identified a conserved serine residue within the active site that mediates recognition (20,21). These studies pinpoint S294 as interacting with N3 and O2 of the cleavage site U to orient the nucleobase in the active site. These positions are involved in Watson–Crick pairing in dsRNA, and thus helix formation of other higher order structures are likely to interfere with cleavage relative to ssRNA or otherwise an unpaired cleavage site U.

Initial studies using short 20mer RNA substrates revealed little sequence preference at B_+1_, where B_+1_ is the nucleotide position 3′ to the scissile phosphate and B_-1_ is the nucleotide located 5′ (B_-1_UB_+1_) (21). However, cyclic phosphate RNA sequencing aimed at identifying Nsp15 cleavage products within mouse hepatitis virus infected bone marrow-derived macrophages showed cleavage at numerous sites throughout the positive strand RNA genome, revealing a B_+1_ preference for UA and CA sequences (22). Importantly, systematic analysis of flanking nucleotide specificity *in vitro* defined the consensus motif for Nsp15 cleavage NU(R > U >> C) for ssRNA (23). Based on available biochemical data and the lack of well-resolved density for flanking bases in cryoEM analyses, Nsp15 does not have an extended binding site for unstructured oligonucleotide substrates (23). This conclusion is further supported by the observation that Nsp15 can accept a minimal 5′-UpG-3′ dinucleotide substrate as well as efficiently cleave short RNA oligonucleotides (24,25). Within an individual RNA substrate containing multiple potential U cleavage sites, rapid cleavage at UA over UC was observed, consistent with purine specificity at B_+1_. However, a second UA site was cleaved much less efficiently within the same RNA substrate (23). Thus, the general preference for B_+1_ can be predictive of cleavage site choice, but other factors involving RNA context can override this aspect of intrinsic Nsp15 specificity.

Early studies of Nsp15 showed robust cleavage of both ssRNA and dsRNA (26,27). However, data from several sources now show that the thermodynamic stability of RNA structure as well as the positioning and sequence context of the cleavage site U play a role in determining Nsp15 alternative substrate specificity (28–30). Changes in GC content that affect RNA structure were shown to alter the cleavage pattern on substrate RNAs (30) and thermodynamically stable RNA structures are protected from Nsp15 cleavage relative to RNAs lacking stable structure (14,28,30). Time courses of cleavage reactions for dsRNA, compared to the individual RNA strands, showed ssRNA reacted more quickly than dsRNA, with different sequence preferences depending on context (29). Cryo-EM structures of SARS-CoV-2 Nsp15 bound to a 52mer dsRNA and to a polyAU RNA reveal a dsRNA-binding site across multiple monomer subunits positioned diagonally in a shallow groove between two subunits of one of the trimers (29,31). These studies further identified a series of residues that interact with the phosphodiester backbone of both strands, which is consistent with overall shape specificity rather than direct interactions with RNA nucleobases. Mutations at the three key interacting regions, termed P1, P2 and P3, disrupt the cleavage of dsRNA with little effect on ssRNA, consistent with a role in the recognition of RNA structure (29).

In this regard, recent structural and biochemical data show that Nsp15 utilizes a base-flipping mechanism to cleave dsRNA to orient the uridine within the active site (29,31). An alanine mutation at the conserved active site residue W333 results in a reduction of ssRNA cleavage but a significantly greater defect in dsRNA, consistent with a role in mediating flipping of the cleavage site U (29). Thus, the current general model for Nsp15 specificity is that it cleaves both ss- and dsRNA effectively if the U is accessible or bulged from the helix. Interestingly, a recent study showed that mutation of cleavage site U residues had minimal effect on RNA binding affinity (28), suggesting that Nsp15 may discriminate between alternative substrates at a step involving a conformational change after initial binding. Intriguingly, the reaction kinetics of a short model substrate, dArUdAdA, showed apparent hysteresis and a cooperative dependence of the observed rate on substrate concentration (18). However, these results are yet to be observed with RNA substrates, although the multiple turnover kinetics of Nsp15 are generally not well characterized. The catalytic EndoU domain of Nsp15 undergoes conformational changes, as revealed by cryo-EM and biophysical methods, that may be linked to substrate binding and ordering of the active site (16,20,32). However, the structure–function relationships that underlie conformational changes and their role in specific steps of the Nsp15 reaction cycle remain to be established.

Initial biochemical studies of Nsp15 showed that Mn^2+^ at mM concentrations is optimal for *in vitro* endoribonuclease activity (26) and resulted in an increase in binding affinity (14,17), yet the Nsp15 active site is devoid of divalent ions (19,25,30). Rather, the Nsp15 active site includes conserved histidine and lysine residues (H235, H250 and K290 in SARS-CoV-2 Nsp15) that are likely to fulfill a common set of catalytic interactions, primarily acid-base and electrostatic catalysis, as in RNase A (13,15–17,20,33–35). The structure of Nsp15 bound to the transition state analog uridine vanadate is consistent with interactions key to these proposed catalytic mechanisms (21). Additionally, a bell-shaped pH-rate profile is observed for multiple RNA substrates in both the presence and absence of Mn^2+^, together with thio effects on the reactive phosphate, strongly support an acid-base catalytic mechanism (25). Interestingly, cryo-EM structures determined at pH 7.5 versus 6.0 show differences that could also be linked to enzyme activity, including the potential for the formation of higher-order structures (18). Higher catalytic activity correlated with a less dynamic open conformation observed at pH 6.0, which was suggested to be similar to the dsRNA complex structure in which the substrate is positioned into the active site (18,29). Thus, protonation states linked to conformation as well as active site ionization may both contribute to Nsp15 reaction kinetics.

Despite significant advances in understanding Nsp15’s contribution to host immune evasion, structure and mechanism, the specificity of the enzyme and how it identifies *in vivo* targets remains mysterious. Here, we report kinetic analyses of a series of model RNA substrates which demonstrate that Nsp15 functions primarily as an RNA structure-specific ribonuclease. The results establish benchmark values for the effects of sequence, location and structure on specificity and represent an essential step toward predictive models of specificity aimed at understanding *in vivo* target selection. We document large differences in *k*_cat_/*K*_m_ between dsRNA, ssRNA and bulged RNA substrates, showing that Nsp15 specificity is dominated by RNA structure and likely to be regulated by factors that alter RNA conformation during the viral life cycle. We provide the first mechanistic insight into how Nsp15 distinguishes between alternative substrates by showing that the chemical step is largely insensitive to structure. Together, these new data define key principles of Nsp15 specificity, demonstrate the intrinsic role of RNA structure in driving cleavage site selection and provide a foundation for deeper investigation of the catalytic cycle and mechanisms of inhibition.

## Materials and methods

### Nsp15 protein expression and purification

Nsp15 from SARS-CoV-2 was overexpressed and purified essentially as described in Huang *et al.* (25), which is based on previous protocols for purification of Nsp15 (35) with modifications. Briefly, Nsp15 protein was expressed as a N-terminal 6xHis tagged polypeptide in T7 Express competent *Escherichia coli* cells (NEB) in Terrific Broth (36) at 37°C with 100 μg/mL ampicillin (25). At an optical density (OD_600_) of 1, protein expression was induced with 0.2 mM Isopropyl β-D-1-thiogalactopyranoside and incubated overnight at 18°C. Cells were harvested the following day by centrifugation, and the cell pellets were stored at −80°C until use. For individual protein purification preparations, cell pellets were resuspended in lysis buffer (50 mM HEPES pH 8.0, 500 mM NaCl, 20 mM imidazole, 5% glycerol, 10 mM β-mercaptoethanol) supplemented with EDTA-free protease inhibitor and lysed by cell disruptor. The resulting lysate was clarified by centrifugation at 40 000 RCF for 1 h at 4°C. The lysate was further clarified by syringe filtration using 25 mm 0.45 μm PES, Glass Fiber Prefilter syringe filters (Foxx Life Sciences). Nsp15 was affinity purified from the lysate using a HisTrap FF (Cytiva) Ni Sepharose column on an AKTA GO chromatography system. After loading the lysate, the column was washed with 50 mM HEPES pH 8, 500 mM NaCl, 10 mM imidazole, 5% glycerol, 10 mM β-mercaptoethanol and Nsp15 was eluted from the resin with 50 mM HEPES pH 8, 500 mM NaCl, 500 mM imidazole, 5% glycerol and 10 mM β-mercaptoethanol. The protein containing fractions were concentrated and buffer exchanged into low salt buffer (50 mM HEPES pH 8, 300 mM NaCl) by using 30 kDa Centricon centrifugation filters (Amicon). The assembled Nsp15 hexamer population was purified by size exclusion chromatography (SEC) on a GE HiLoad 16/600 Superdex 200 pg column in SEC buffer (50 mM HEPES pH 8, 300 mM NaCl, 1 mM β-mercaptoethanol and 5% glycerol). Hexamer species were concentrated by Millipore 30 kDa centrifuge filters and the resulting enzyme stocks were stored at −80°C until further use.

### Multiple turnover reactions

RNA oligonucleotide substrates were obtained commercially (Horizon Discovery) as solid phase synthesis products with the sequences and the 5′ and 3′ fluorescent labels shown in Table 1. The RNA secondary structure and stability for each of the synthetic RNA substrates were analyzed using RNAFOLD (37), which yielded the structures and calculated folding free energies described in the Results. The 1S PL (plate reader) substrates 1S_ss UA_PL, 1S UC_PL and 1S UG_PL contain a Cy3 fluorophore on the 5′ end and an additional BHQ-2 modification on the 3′ end. This configuration results in quenching in the intact precursor RNA and a fluorescence-on signal upon product formation. We similarly used an increase in the fluorescence emission upon cleavage of the 1S_UG/UC and PUN_ss28 substrates to measure reaction kinetics for these substrates. The 6S_ss22 and PUN_ss28 RNA substrates are modified at either terminus with Cy5 or fluorescein (FL) to allow the identification of the corresponding 5′ and 3′ Nsp15 cleavage products. All the synthetic substrates were obtained as 2′-bis(2-acetoxyethoxy)methyl protected RNAs that were deprotected according to the manufacturer's protocol, aliquoted and stored at −20°C until use.

**Table 1.** Sequence of synthetic RNA substrates

Substrate RNA	Sequence
1S_ds	5′Cy3-GAAAGCCAGCGAAAGCUGGCAAGA-3′
1S_loop	5′Cy3-GAAAGCCGCGUAAGCGGCAAGA-3′
1S_bulge	5′Cy3-GAAAGCCGCGAAAGCUGGCAAGA-3′
1S_ssUG	5′Cy3-GCCCAAAAUGGACCGAGCGAG-3′
1S_ssUA_PL	5′Cy3-GCCCAAAAUAGACCGAGCGAG-BHQ2-3′
1S_ssUC_PL	5′Cy3-GCCCAAAAUCGACCGAGCGAG-BHQ2-3′
1S_ssUG_PL	5′Cy3-GCCCAAAAUGGACCGGGCGGG-BHQ2-3′
2S_ss21UG/UC	5′Cy3-ACCCAAAAUGGUCCGGGCGGG-3′
2S_ss17UG/UC	5′Cy3-AAAAUGGUCCGGGCGGG-3′
2S_ss20UG/UA	5′Fl-AAAAAGAUGGCUACGACGGG-3′
6S_ss22	5′Cy5-AAGUAGUAGUAGUAGUAGUAGA-Fl-3′
PUN_ss28	5′Fl-UUUUUUUUUUGUCAUUCUCCUAAGAAGC-Cy5-3′

To determine the *k*_cat_/*K*_m_ for Nsp15 cleavage reactions were performed using enzyme concentrations of 25–50 nM and 250 nM–20 μM of the appropriate RNA oligonucleotide substrate. Reaction buffer typically contained 50 mM HEPES (pH 7.0), 100 mM KCl, 5 mM MnCl_2_ and 1 mM DTT at 25°C with the divalent ion type and concentration, and the reaction pH adjusted as described in the 'Results' section and figure legends. Structured substrates were folded by heating to 95°C for 3 min and slowly cooling to room temperature.

To measure Nsp15 kinetics by plate reader a 50 nM enzyme stock in reaction buffer (50 mM HEPES (pH 7.0), 100 mM KCl, 5 mM MnCl_2_ and 1 mM DTT) was distributed equally in 20 μL aliquots on a Costar 96 well plate. Substrate stocks were prepared from 250 nM to 20 μM in the same buffer. One well was prepared with 1× Nsp15 reaction buffer alone to obtain a blank and another was prepared with the highest concentration of the respective substrate alone to set the gain on the plate reader detector before starting measurements and to serve as a negative control. After incubation for 15 min, 30 ul of substrate at different concentrations was injected to 30 ul of constant enzyme concentrations using a multichannel pipette. The final volume per well is 60 ul. The change in fluorescence intensity was recorded using a BMG Clariostar plate reader for each well, which was used to determine the reaction amplitude and rate. The excitation was measured at 530–20 nM and the emission was measured at 580–30 nM for every 10 s till 5000 s at 25°C. Number of flashes per well were set to 20.

To measure Nsp15 kinetics using gel electrophoresis to resolve precursor and products RNAs, reactions were initiated by mixing equal volumes of enzyme and substrate, and aliquots from the reaction were taken at selected time points. Reactions were stopped by adding twice the volume of gel loading dye (80% formamide, 10 mM EDTA, xylene cyanol). The precursor and product RNAs were resolved on 20% denaturing urea polyacrylamide gels for 2 h at 120V. The gel images were scanned using a Typhoon Phosphorimager (Cytiva) and quantified using ImageJ software to determine the relative intensity of substrate (S) and product (P) bands. The fraction of product formed (F = P/(S + P)) was calculated based on the band intensities and plotted versus time.

### Single turnover reactions

To measure the rate constant for catalysis (*k*_c_) for the substrates reported in Table 2 , we used single turnover reactions under saturating enzyme conditions (25). The RNA substrate and Nsp15 enzyme concentrations were 50 and 500 nM, respectively. The enzyme and substrate at twice the final concentration were incubated separately in reaction buffer (50 mM HEPES (pH 7.0), 100 mM KCl, 5 mM MnCl_2_ and 1 mM DTT) for 10 min at 25°C before initiating the reaction by mixing. Time points were collected by stopping the reaction in aliquots with 1:2 volume of gel loading dye (80% formamide, 10 mM EDTA, xylene cyanol). The reaction time points were resolved on 20% denaturing urea polyacrylamide gels for 2 h at 120V. The gels were scanned using a Typhoon Phosphorimager (Cytiva) and quantified using ImageJ software to determine the relative intensity of substrate (S) and product (P) bands.

**Table 2.** Multiple and single turnover kinetics of Nsp15 cleavage of model RNA substrates

Substrate RNA	*k*_cat_/*K*_m_ (x 10^6^ M^−1^s^−1^)	*k*_c_ (s^−1^)
1S_ds		0.0010 ± 0.0004
1S_loop		0.00057 ± 0.0004
1S_bulge	0.079 ± 0.002	0.0014 ± 0.0001
1S_ssUG_PL	0.009 ± 0.001	0.0017 ± 0.0001
1S_ssUA_PL	0.016 ± 0.003	0.005 ± 0.001
1S_ssUC_PL	0.0039 ± 0.0003	0.0024 ± 0.0005
2S_ss21UG/UC	0.017 ± 0.004^a^	0.0029 ± 0.0019
PUN_ss28	0.34 ± 0.02^a^	0.006^b^

^a^Because the 2S_ss21UG/UC and PUN_ss28 substrates contain multiple U cleavage sites the reported initial rate represents the sum of the individual initial rates for all of the sites independent of product inhibition.

^b^From reference (25)

### Data analysis

**Figure 1.**
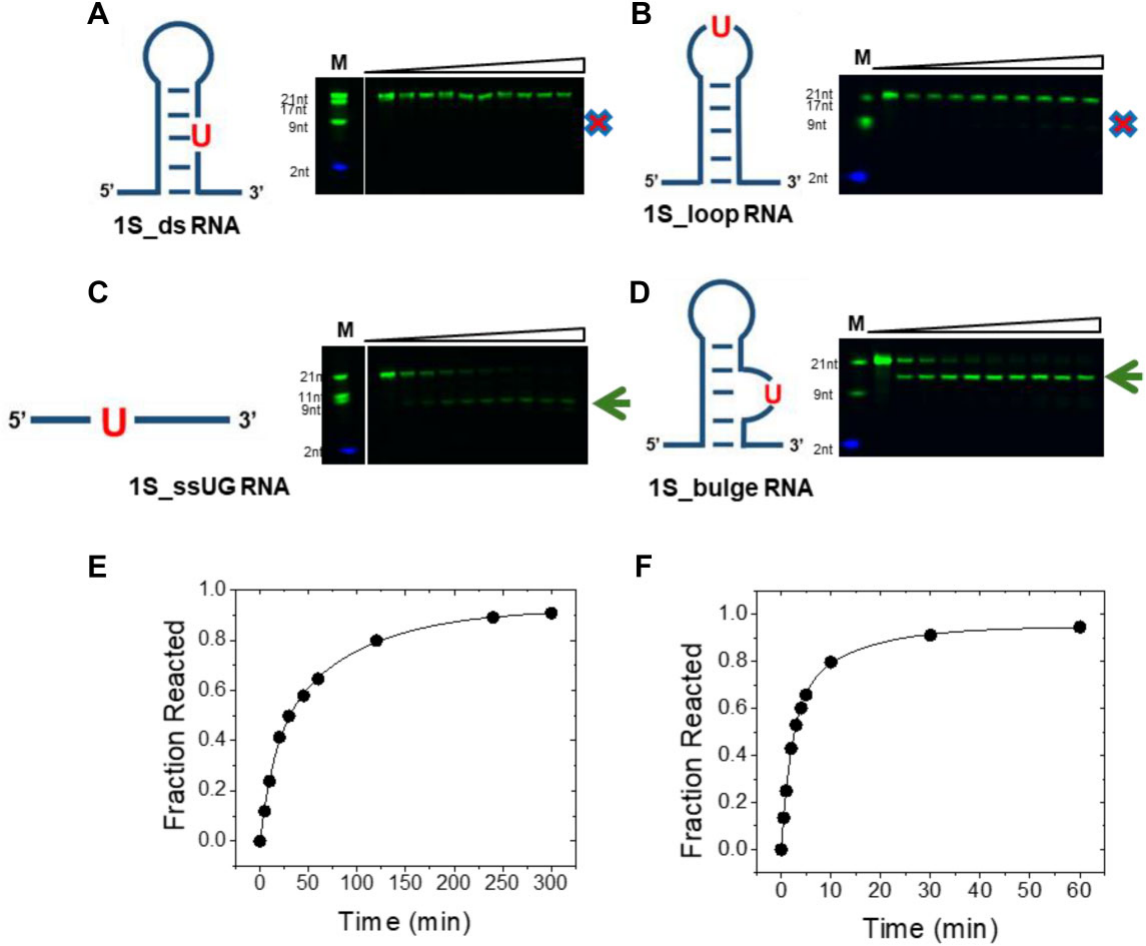
Multiple turnover kinetics of Nsp15 cleavage of dsRNA, ssRNA, loop and bulged RNA substrates. (**A**) Substrate sequence and structure of 1S_ds RNA substrate with the position of the single U residue highlighted. Below is shown the PAGE analysis of reaction time course. (**B**) The sequence and structure of 1S_loop RNA substrate with the position of the single U residue highlighted. PAGE analysis of reaction time course is shown to the right. (**C**) Substrate sequence and structure of 1S_ss RNA substrate with the position of the single U residue highlighted. Below is shown the PAGE analysis of reaction time course where the site of cleavage is indicated by an arrow. (**D**) Substrate sequence and structure of 1S_bulge RNA substrate with the position of the single U residue highlighted. To the right is shown the PAGE analysis of reaction time course where reaction products are indicated as in panel C. (**E**) and (**F**) Time dependence of product formation of Nsp15 cleavage product of 1S_ssUG RNA and 1S_bulge RNA, respectively. The time course data are fit to a double exponential function which is used to illustrate the progress of the reaction. However, as described in 'Materials and methods' section, the initial rate (*v*_ss_) was used to determine *k*_cat_/*K*_m_. An X marks the position of expected cleavage. An arrow indicates product formation.

**Figure 2.**
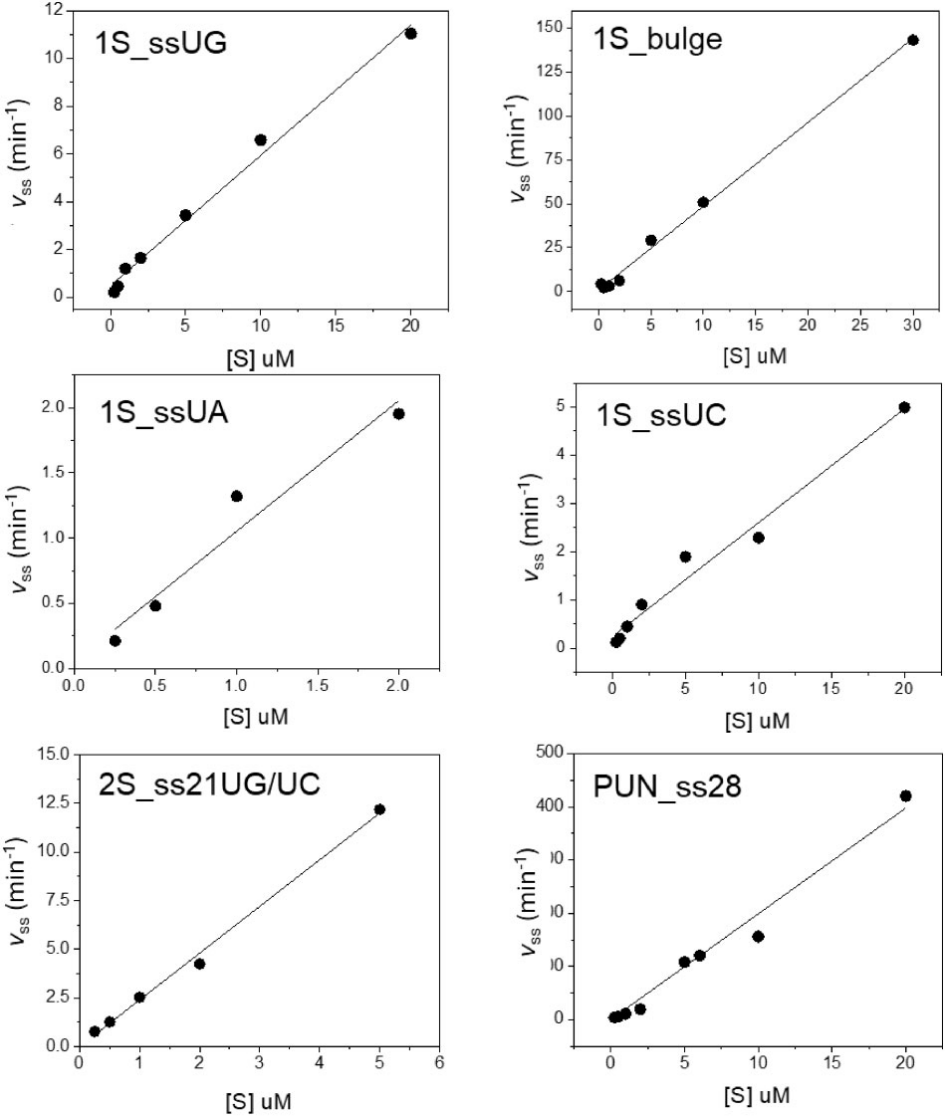
Determination of *k*_cat_/K_m_ for Nsp15 cleavage of model RNA substrates. The observed steady state initial rate (*v*_ss_) was determined over a range of RNA substrate concentrations (0.25–20 μM) as described in 'Materials and methods' section. The identity of the substrate used in the reaction is shown in the upper left corner of each set of axes. Data were fit to a linear equation and the *k*_cat_/*K*_m_ values are shown in Table 2. For 1S_ssUG_PL, 1S_ssUA_PL, 1S_ssUC_PL and 2S_ss21UG/UC, the data were also fit to the Michaelis–Menten equation (equation 1) shown in Supplementary Figure S2, and the *k*_cat_/*K*_m_ values determined by the two approaches are compared in Supplementary Table S2.

For the determination of *k*_cat_/*K*_m_, the fluorescence-based plate reader assay was used to determine reaction rates. Measuring the rate for the 1S_bulge substrate by fluorescence was problematic due to the instrument dead time and sensitivity; therefore, rates were quantified from gel data using phosphorimager analysis. We examined the systematic error between the two methods by comparing the results for 1S_ssUG, 1S_ssUG/UC and PUN_ss28 from Figures 1, 2 and 7 to the plate reader data obtained under the same conditions. The initial rates for 1S_ssUG, 1S_ssUG/UC and PUN_ss28 estimated by fitting data obtained by the gel assay to a single exponential function were 0.24 min^−1^ (R^2^ 0.98), 0.53 min^−1^ (R^2^ 0.95) and 23 min^−1^ (R^2^ 0.96), respectively. The observed rate constants for 1S_ssUG, 1S_ssUG/UC and PUN_ss28 determined using fluorescence under the same conditions as the corresponding gel data were 0.448 ± 0.003 min^−1^, 1.3 ± 0.01 min^−1^ and 11.0 min^−1^ ± 0.4 min^−1^, respectively (Supplementary Table S1). Thus, the difference in the observed rate determined using the two methods may be up to 2-fold lower for the gel method. Importantly, these limits to precision are smaller than the *ca*. 10-fold difference in the multiple turnover kinetics for 1S_bulge versus other substrates is outside the range of systematic error between the assays.

To maximize the number of points and precision for initial rate measurements multiple turnover kinetic data for the determination of *k*_cat_/*K*_m_ were primarily obtained using the fluorescence-based plate reader assay. Example data demonstrating fitting results are shown in Supplementary Figure S1 for 1S_ssUG_PL RNA. The observed initial rates (*v*_obs_ nM/s) were calculated by fitting the first 10% of product formation (typically 20–30 points) estimated from the reaction amplitude to a linear function. Supplementary Figure S1 also shows that equivalent results for *v*_obs_ could be obtained by fitting the entire fluorescence progress curves to a single exponential function (equation 2, below). The *v*_obs_ values were normalized to the total enzyme concentration of the reaction to yield the steady state multiple turnover rate (*v_ss_* s^−1^). The average *v_ss_* values from at least three repeats obtained using substrate concentrations 0.25–30 μM were determined (Supplementary Table S2), and these data were fit to a linear function to give the *k*_cat_/*K*_m_ (M^−1^s^−1^) reported in Table 2.

We also analyzed the data for these substrates using the Michaelis–Menten equation.


(1) \begin{equation*}{{v}_{ss}} = \frac{{{{k}_{cat}}\left[ S \right]}}{{{{K}_m} + \left[ S \right]}}\end{equation*}

The values for *k*_cat_/*K*_m_ that are obtained using the two approaches are compared in Supplementary Table S2. The values correspond for both methods providing a check on accuracy; however, greater fitting error for equation 1 was observed relative to the values obtained from linear fitting, which are therefore reported in Table 2 and allow comparison between substrates using a single methodology.

For determination of *k*_c_ from single turnover kinetic data, the fraction of product formed (F = P/(S + P)) was calculated based on the band intensities from phosphorimager analysis and plotted versus time. The data were fit to a single exponential function,


(2) \begin{equation*}F = A{{e}^{ - {{k}_c}t}} + B\end{equation*}

Where *A* is the amplitude of the exponential and *B* is the initial background signal. Depending on the appearance of non-random residuals indicating a poor fit to equation 2, the data were fit to a double exponential equation,


(3) \begin{equation*}F = {{A}_1}{{e}^{ - {{k}_1}t}} + {{A}_2}{{e}^{ - {{k}_2}t}} + B\end{equation*}

In which *k*_1_ and *A*_1_ are the rate constant and amplitude of the fast reaction phase, and *k*_2_ and *A*_2_ are the corresponding rate constant and amplitude of the slow reaction phase.

## Results

### Helical structure can contribute orders of magnitude to Nsp15 specificity as either a determinant or an anti-determinant

To quantify the contribution of the structural context surrounding the U cleavage site on Nsp15 specificity, we compared the multiple turnover rates measured at limiting substrate concentration for a series of model RNA substrates. The ability of an enzyme to discriminate between alternative substrates is proportional to the ratio of their concentrations and their *k_cat_*/*K_m_* values (38–40). Accordingly, estimating the quantitative effects of structure and sequence on the second-order rate constant *k*_cat_/*K*_m_, or specificity constant, is key to developing a useful model that can describe and predict which site or sites within an RNA will be cleaved and how fast (41).

A set of substrates was designed that have similar sequences and position a single U residue within different RNA structure contexts (Figure 1 and Table 1). The 1S_ds substrate is a 25 nucleotide RNA that positions a single U within a six-base pair helix capped by a highly stable GNRA tetraloop with the sequence GAAA (42). The RNA secondary structure stability was analyzed using RNAFOLD(37), which yielded a single secondary structure model with a free energy of −14.66 kcal/mol. The 1S_loop RNA positions the single U in the variable (N) position of the GNRA tetraloop and is predicted to fold into the same structure as 1S_ds with a similar folding free energy of −16.22 kcal/mol. The 1S_bulge RNA has the U cleavage site located as a single nucleotide bulge within the six-base pair stem on the 3′ side of the stem loop and has a free energy of −8.29 kcal/mol. In contrast, the 1S_ssUG RNA sequence minimizes consecutive pairs and is predicted to form an ensemble of structures with a free energy of −1.92 kcal/mol. All four substrates contain a 5′ Cy3 modification to visualize the conversion of precursor RNA to the 5′ product fragment, allowing the quantification of reaction kinetics as described in 'Materials and methods' section.

Analysis of the time courses of product formation for Nsp15 cleavage of the 1S_ssUG, 1S_ds, 1S_loop and 1S_bulge substrates show that only the 1S_bulge and 1S_ssUG RNAs react to near completion during the time course of the reaction. In contrast, very little product is detected for the 1S_loop RNA even after prolonged incubation, and the 1S_ds substrate did not form detectable product under the conditions tested. The migration of the products observed for the 1S_ssUG, 1S_loop and 1S_bulge RNA substrates relative to synthetic oligonucleotide standards are consistent with Nsp15 catalyzing strand cleavage 3′ of the unique U residue (Figure 1). The rates of cleavage of 1S_ssUG and 1S_bulge are both sufficient for the reaction to proceed to completion within 1 h; however, the observed rate is clearly faster for 1S_bulge. To estimate the relative *k*_cat_/*K*_m_ for these substrates and others listed in Table 2, the observed initial rate was determined over a range of substrate concentrations (0.25–30 μM). The data for each substrate were determined using a linear function as shown in Figure 2 to give the *k*_cat_/*K*_m_ (M^−1^s^−1^) values reported in Table 2. The data for 1S_ssUG_PL, 1S_ssUA_PL, 1S_ssUC_PL and 2S_ss21UG/UC were also evaluated using the Michaelis–Menten equation (equation 1) (Supplementary Figure S2). The lack of data at high concentrations results in poorly constrained values for *k*_cat_ and *K*_m_; nonetheless, the estimated values for *k*_cat_/*K*_m_ correspond with the results from linear fitting (Supplementary Table S2), which was used for comparison between substrates.

As expected, the *k*_cat_/*K*_m_ for 1S_bulge is significantly higher at 0.079 ± 0.002 × 10^6^ M^−1^s^−1^ compared to 0.0090 ± 0.001 × 10^6^ M^−1^s^−1^ for 1S_ssUG. For comparison, a *k*_cat_/*K*_m_ of 0.015 × 10^6^ M^−1^s^−1^ was measured for Nsp15 cleavage of a UpG dinucleotide (25). These values further contrasts with the observed *k*_cat_/*K*_m_ of 3.5 × 10^6^ M^−1^s^−1^ for RNase A cleavage of UpA and 1 × 10^6^ M^−1^s^−1^ for RNase T1 cleavage of GpU (43,44).

Based on these results, several key principles that direct Nsp15 specificity beyond U preference are apparent. The presence of a stable helical structure acts as a strong negative anti-determinant and essentially blocks Nsp15 recognition. While cleavage of 1S_ssUG RNA is readily observed, product formation is not detected over the same time course for 1S_ds. In contrast, helical structure for 1S_bulge acts as a significant positive determinant for specificity, resulting in a *∼*9-fold greater relative *k*_cat_/*K*_m_ compared to the 1S_ssUG substrate, which lacks helical structure. Only a small (<10%) amount of product is formed for the 1S_loop RNA substrate over the time course which corresponds to a rate decrease of *ca*. 10-fold relative to 1S_ssUG that reacts to 10% conversion within 2 min. No product is observed for the reaction of 1S_ds RNA consistent with even slower reaction kinetics than 1S_loop. Thus, RNA structure can result in multiple orders of magnitude differences in relative Nsp15 cleavage rate and can act as a negative anti-determinant to decrease *k*_cat_/*K*_m_ or as a positive determinant compared to unstructured ssRNA.

### B_+1_ preference guides competition between alternative cleavage sites but observed rates depend on local sequence context

**Figure 3.**
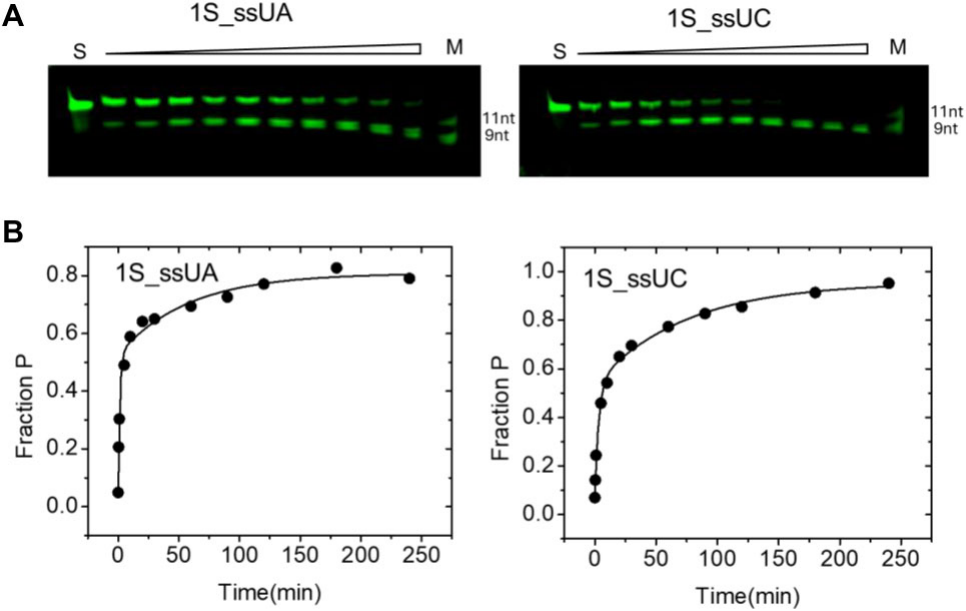
Analysis of multiple turnover reactions of 1S_ssUA and 1S_ssUC cleavage by Nsp15. (**A**) Time courses of product formation for multiple turnover reactions of 1S_ssUA (left) and 1S_ssUC (right) at 50 nM Nsp15 concentration and 500 nM substrate RNA in standard reaction buffer with 5 mM Mn^2+^ at pH 7. The unreacted substrate (**S**) and marker oligonucleotides (11nt, 9nt) are indicated above in the appropriate lanes. (**B**) Plots showing the fraction product formed (Fraction P; [P]/([P]+[S]) versus time (min). The kinetics or product formation quantified from the gel data are fit to a double exponential function that is used to illustrate the progress of the reaction. However, as described in 'Materials and methods' section, the initial rate (*v*_ss_) was used to determine *k*_cat_/*K*_m_.

Previous studies show that Nsp15 prefers purines over pyrimidines in the B_+1_ position, and Nsp15 is also reported to efficiently process substrates with polyU sequences (22,23). In order to make quantitative comparisons between different sequence and structure contexts, we measured the effect of B_+1_ on the relative *k*_cat_/*K*_m_ in the context of the 1S_ssUG substrate (Figures 2 and 3). The results are consistent with previous studies and, importantly, they provide necessary information for evaluating the relative contributions of sequence to that of structure. The results show that the preference for UA relative to UG is slight, with only ∼2-fold slower rate constant for G at B_+1_, while the rate of 1S_ssUC_PL cleavage is *∼*4-fold slower compared to 1S_ssUA_PL (Table 2).

**Figure 4.**
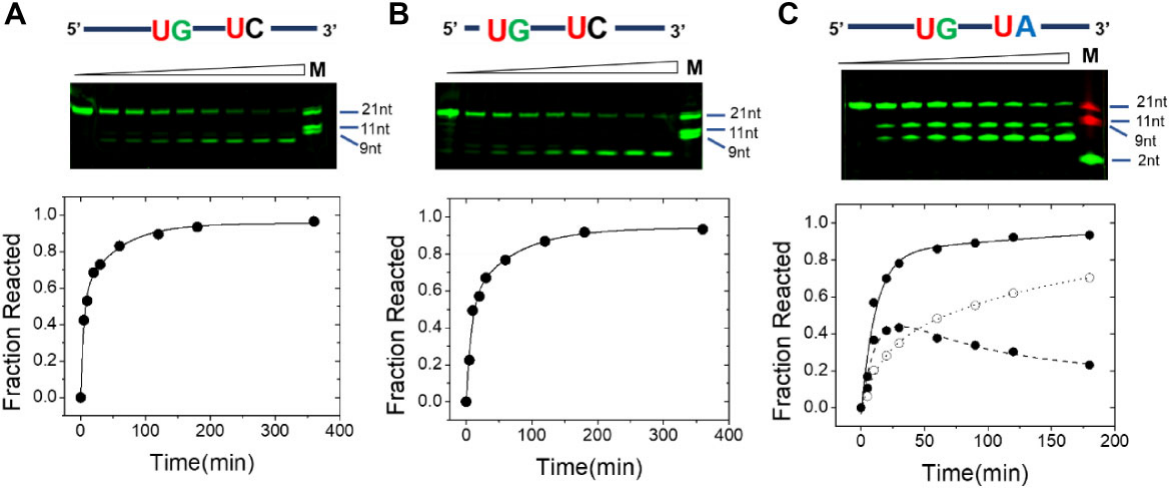
Comparison of the multiple turnover kinetics of ssRNA substrates containing competing U cleavage sites. (**A**) Analysis of Nsp15 cleavage of 2S_ss21UG/UC. The products of a time course of multiple turnover cleavage of 500 nM RNA and 50 nM Nsp15 were resolved by denaturing PAGE. A plot of the fraction of substrate converted to product (Fraction Reacted) versus time for conversion of 2S_ss21UG/UC resulting from cleavage at the UG site is shown below. The data are fit to a double exponential function to approximate the behavior of the entire multiple turnover time course. (**B**) Time course of Nsp15 cleavage of 2S_ss17UG/UC under the same conditions as panel A. The plot of fraction reacted versus time shown below quantifies the kinetics of accumulation of the substrate to form the UG cleavage product. (**C**) Time course of product formation generated by multiple turnover cleavage of 2S_ss20UG/UA under the same conditions as panels A and B. The plot shows the kinetics of total product formation resulting from cleavage at both the UG and UA sites (filled circles, solid line) and the accumulation of the UG cleavage product (open circles, dashed line) and the accumulation and subsequent decay of the UA cleavage product (filled circles, dashed line).

To further outline the effects of local sequence and structure on Nsp15 specificity, we asked whether the relative rates for B_+1_ specificity were predictive of cleavage site selection in the context of a model substrate with competing Nsp15 cleavage sites (Figure 4). The 2S_ss21UG/UC substrate is a modified version of the 1S_ssUG substrate, which contains two potential cleavage sites, UG and UC dinucleotides, positioned near the center of a 21 nucleotide ssRNA (Table 1). Under initial rate conditions, the relative rate of product formation resulting from cleavage at UG versus UC is expected to reflect the relative *k*_cat_/*K*_m_ for the two sites (0.009 versus 0.0039 × 10^6^ M^−1^s^−1^, or a ratio of *∼*2-fold). However, only UG cleavage is observed, while a larger product of 12 nucleotides due to UC cleavage in the 2S_ss21UG/UC RNA is not observed. Since the substrate RNA is labeled at the 5′ end, the products of secondary cleavage of the 3′ product formed after initial cleavage at UG are not detectable. Quantification of the PAGE data shows that UG cleavage accounts for all the substrate consumed in the reaction. Thus, the quantitative preference of Nsp15 for UG versus UC is generally predictive of the pattern of products formed at steady state. A truncated version of this substrate in which the UG dinucleotide is located 5 nucleotides from the 5′ end (2S_ss17UG/UC) shows essentially identical kinetics (Figure 4). The single cleavage product observed for Nsp15 cleavage of 2S_ss17UG/UC is smaller than the product observed for 2S_ss21UG/UC, consistent with migration relative to oligonucleotide size standards consistent with UG cleavage.

To further investigate the competition between alternative sites, we examined the kinetics of Nsp15 cleavage of 2S_ss20UG/UA, which contains two fast reacting UR sites separated by three nucleotides. PAGE analysis of the cleavage products shows initially there are similar amounts of product formed due to cleavage at both sites, consistent with very similar *k*_cat_/*K*_m_. The larger cleavage product consistent with Nsp15 cutting at UA accumulates somewhat more rapidly early in the reaction (<25 minutes), while at longer incubation, the smaller cleavage product resulting from UG cleavage accumulates. This pattern of product formation is consistent with UA having a slightly larger *k*_cat_/*K*_m_ compared to the UG site. The decrease in the larger UA cleavage product later in the reaction coincides with the continued increase in the shorter product arising from UG cutting, consistent with re-cleavage by Nsp15. The pattern of relative rates of cleavage in the context of multi-site substrates is thus consistent with the hierarchy of nucleobase preference at B_+1_ determined in the 1S_ssUG RNA context.

### Nsp15 catalysis is comparatively insensitive to substrate sequence and structure

Previous biophysical and structural studies of Nsp15 provide evidence for conformational changes involving the catalytic EndoU domain, which may be linked to substrate binding or catalysis (16,20,45). Since the *k*_cat_/*K*_m_ for a particular substrate reflects all steps up to and including the first irreversible step in the multiple turnover reaction, it is important to understand the extent to which individual steps are rate limiting (46). To compare the rate of catalysis (*k*_c_) for the different model RNA substrates, we used single turnover kinetics at saturating enzyme concentrations which measures the rate of conversion of ES to EP (47,48).

**Figure 5.**
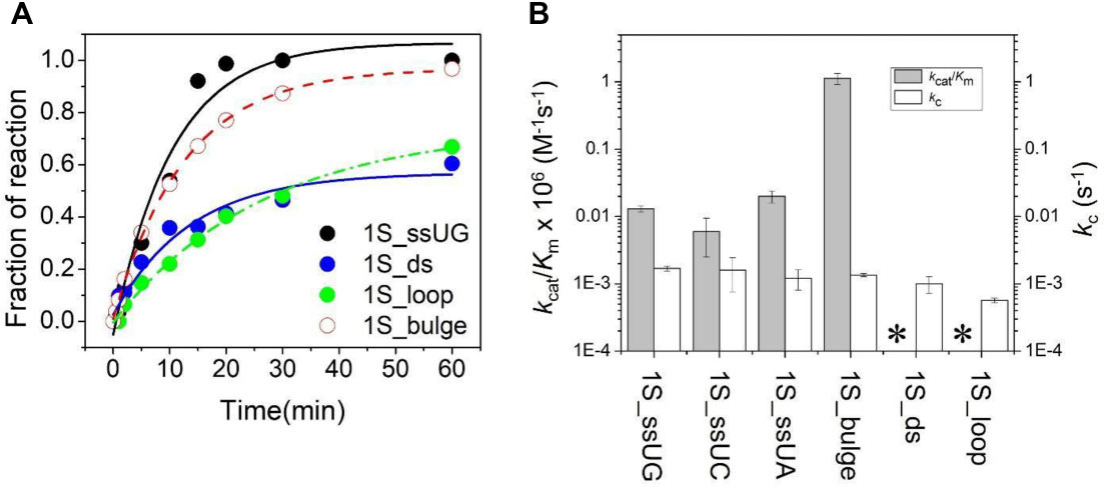
Comparison of single turnover (*k*_c_) and multiple turnover (*k*_cat_/*K*_m_) kinetics for Nsp15 cleavage of model substrates. (**A**) Plot of product formation versus time for Nsp15 single turnover cleavage of model structured RNA substrates 1S_bulge (solid line), 1S_ssUG (dashed line), 1S_ds (solid line) and 1S_loop (dashed line). The data are fit to a single exponential function (equation 2). (**B**) Histogram comparing the effects of RNA structure variation on Nsp15 catalysis (*k*_c_) (open columns) and specificity (*k*_cat_/*K*_m_) (filled columns). The *k*_cat_/*K*_m_ values for 1S_ds and 1S_loop were too low to measure and are therefore indicated by asterisks.

The 1S_bulge and 1S_ssUG substrates were cleaved with essentially identical rate constants (0.0014 s^−1^ versus 0.0017 s^−1^) (Figure 5 and Table 2). The 1S_loop substrate is converted to product approximately 2–3-fold more slowly compared to 1S_bulge and 1S_ssUG. There is little effect of variation at the B_+1_ nucleobase as the differences between the 1S_ss UA, UG and UC are all less than 2-fold. As shown above, the *k*_cat_/*K*_m_ of 1S_ds was so low compared to the other three model substrates that essentially no product was detected over the same time course. However, under single turnover conditions in which the binding step is rapid compared to catalysis, the 1S_ds substrate is cleaved with kinetics comparable to 1S_loop (0.001 s^−1^ versus 0.00057 s^−1^). Interestingly, the kinetics of 1S_ds RNA are biphasic in that only *ca*. 50% of the initial substrate population reacts with fast kinetics while the remaining substrate reacts much more slowly over the time course of the experiment. This result is consistent with two slowly exchanging or non-exchangeable conformations of the substrate, one that is reactive and the other essentially unreactive over the time course of the experiment. Alternatively, it is possible that the accumulation of the cleaved 1S_ds fragments are able to rebind the enzyme and result in product inhibition. Nonetheless, the rate at which Nsp15 cleaves the bound RNA substrate, *k*_c_, varies less than 4-fold for the different model structures and sequences analyzed here. In contrast, the effect of variation in RNA structure on *k*_cat_/*K*_m_ is much more profound, ranging over several orders of magnitude, consistent with discrimination between alternative substrates occurring at a binding step or conformational change before catalysis.

### The position of the target U within an unstructured RNA has minimal effect on Nsp15 specificity

**Figure 6.**
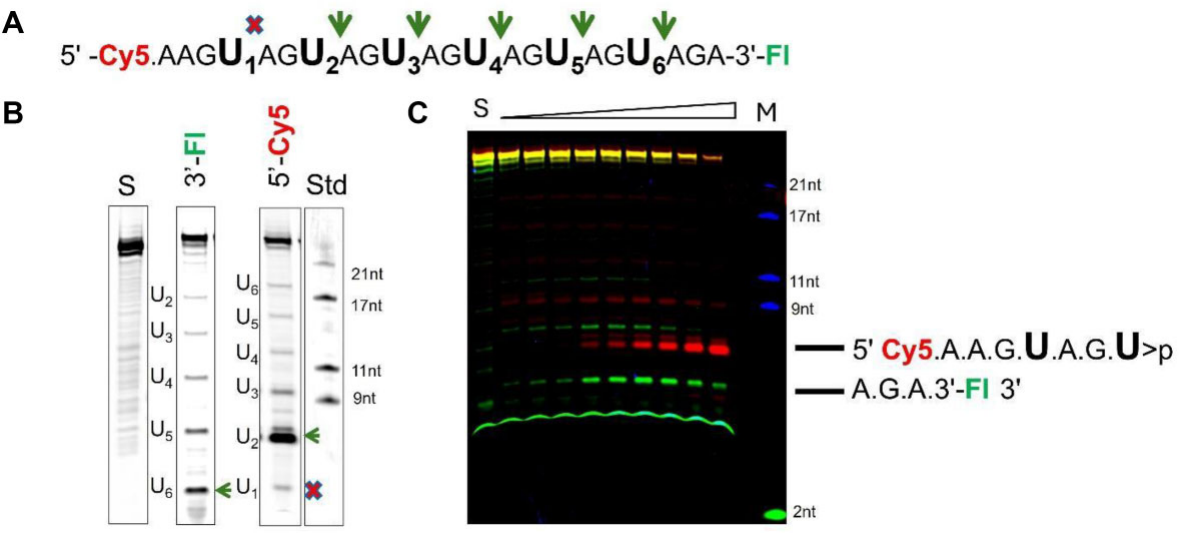
Nsp15 multiple turnover cleavage within a ssRNA with six competing sites. (**A**) Sequence of 6S_ss22 RNA with the U residues that are sites of potential Nsp15 cleavage highlighted in bold and numbered from the 5′ end. The observed cleavage sites are indicated by arrows and the lack of cleavage at U_1_ is denoted by an X. (**B**) Comparison of the distribution of initial products formed as detected using the 3′-FL label or 5′-Cy3 labels. The products corresponding to cleavage at sites U_2_-U_6_ are indicated. (**C**) Merged Cy3 and Fl channels for an entire time course of 6s_ss22 cleavage by Nsp15. The 5′ product corresponding to cleavage at the U_2_ site and 3′ product corresponding to cleavage at the U_6_ site are shown on the right.

Recent structures of Nsp15 bound to dsRNA are consistent with extended interactions with helical structure consistent with a positive determinant for bulged U substrates like 1S_bulge RNA (29,31). However, it is not clear whether ssRNA can form extended contacts with Nsp15 to influence cleavage rates, resulting in the position of U residues within an oligonucleotide context contributing to alternative substrate recognition. To test whether the location of the target U residue within the context of an otherwise unstructured RNA impacts cleavage site selection, we designed a substrate containing six UpA cleavage sites (sites U_1_-U_6_) in the context of a repeating AGUAG sequence (Figure 6). The 5′ and 3′ ends of the 6S_ss22 RNA (Table 1) are labeled with different fluorophores (5′Cy5 and 3′Fl) so that the initial rate of cleavage at the six different sites (U_1_-U_6_) could be compared. Under multiple turnover conditions, the pattern of cleavage at early time points can be used to quantitatively compare the effect of location within the ssRNA sequence. Re-cleavage of the initial products will produce progressively smaller RNAs, and the length of these final products can provide information about whether the proximity of the target U to the RNA 5′ and 3′ termini alters the cleavage rate.

The initial products of 6S_ss22 cleavage are five distinct products that are detected by both the 3′ Cy5 and 3′ FL fluorescent labels (Figure 6). This result is consistent with similar initial rates of Nsp15 cleavage at the five sites and therefore similar *k*_cat_/*K*_m_ values regardless of their location in the linear 6S_ss22 RNA sequence. The observation of only five cleavage products detected by both 5′ and 3′ end labels, however, indicates that one of the six sites is refractory to Nsp15 cleavage. Continued incubation results in the initial five cleavage products becoming substrates that undergo secondary cleavage, resulting in progressively smaller fragments, ultimately resulting in a 3′ cleavage product consistent with cleavage at the U_6_ site (Figure 6C). Thus, Nsp15 can cleave substrates with at least 3 nucleotides of the U cleavage site. The migration of the final 5′Cy5 labeled product is consistent with a fragment of 7 nucleotides resulting from Nsp15 cleavage at the U_2_ site but not at U_1_. The fact that this 5′ cleavage product does not undergo further cleavage by Nsp15 is consistent with the involvement of 5′ flanking sequences for recognition. However, we cannot exclude the possibility that the presence of a 5′ Cy5 modification interferes with Nsp15 recognition at the U_1_ site. A previous study showed, however, that a Cy5 located 2 nucleotides 5′ to a U cleavage site is a substrate for Nsp15, albeit at high (> 1 μM) enzyme concentration (24). Notably, the *k*_cat_/*K*_m_ for a UpG dinucleotide substrate is similar to the value for 1S_ssUG (0.015 × 10^6^ versus 0.009 × 10^6^ M^−1^s^−1^; Table 2). This result further argues that there is little functional contribution from flanking nucleobases, at least in the context of these simple model substrates. However, these results do not exclude the potential for sequence specific effects that are missing in the substrates used here, nor the effect of flanking sequences and sequence length differences on the formation of higher order structure.

### Evidence that local sequence context can override B_+1_ specificity

**Figure 7.**
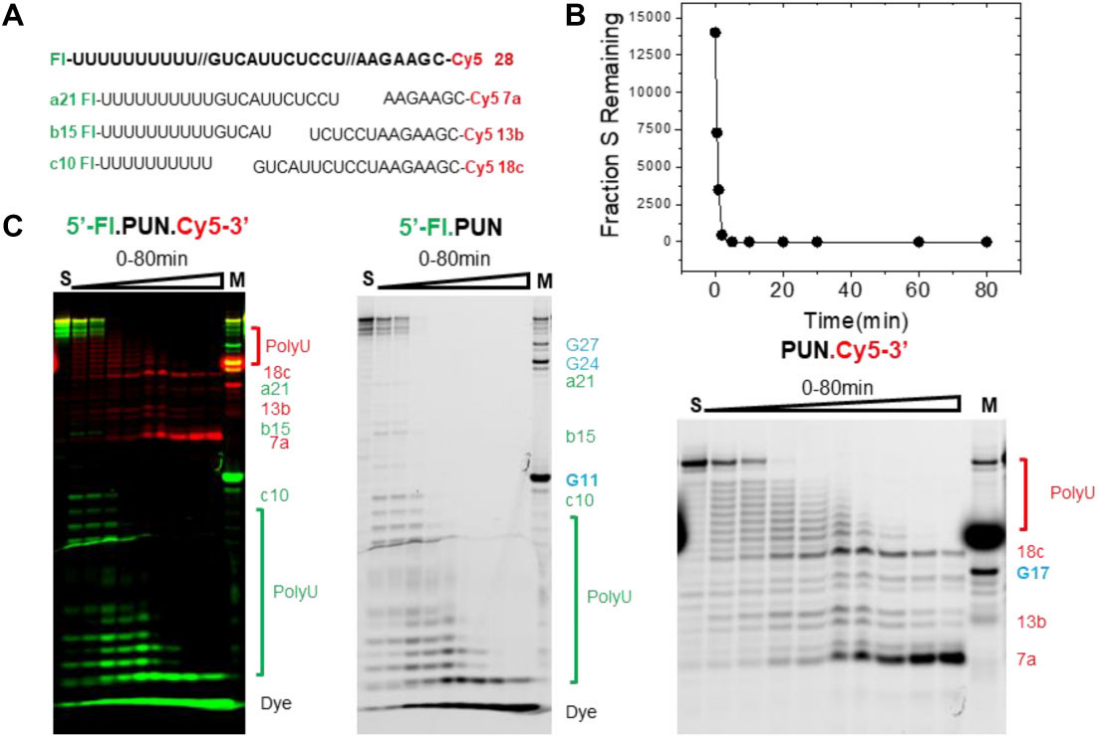
Analysis of Nsp15 multiple turnover cleavage of PUN_ss28 RNA. (**A**) The sequence of PUN_ss28 is shown and the 5′ fluorescein modification (Fl) and 3′ Cy5 modification are indicated. The sequences of major 5′ cleavage products observed in the Fl channel (Green) are shown below and labeled a21, b15 and c10, respectively. Similarly, the sequence of the major cleavage products observed in the Cy5 channel (Red) are 7a, 13b and 18c. (**B**) Kinetics of PUN_ss28 RNA multiple turnover cleavage in reactions containing 0.5 μM PUN_ss28 RNA, 50 nM Nsp15 and standard reaction buffer with 5 mM Mn^2+^. The data were fit to a single exponential (equation 2) to show reaction progress. (**C**) Analysis of the products of PUN_ss28 cleavage by Nsp15 using PAGE. The panel labeled Fl.PUN.Cy5-3′ shows the gel with the Fl and Cy5 channels merged, and major cleavage products are indicated to the right of the gel. Products resulting from cleavage within the 5′ polyU leader for Fl and Cy5 channels are indicated by brackets. The separate gel image of both the channels is also shown in grayscale. The major products of the RNase T1 ladder for both the channels are indicated in blue. The major RNase T1 products G27, G24, G11 and G17 are indicated on the respective gels of respective channels. The gel showing the PUN.Cy5-3′ channel is scaled to show more clearly the resolution of products resulting from cleavage near the 5′ end and the similar intensities for polyU, UG and UA cleavage events.

Given the apparent lack of sequence context on Nsp15 cleavage specificity in unstructured RNA, we further examined whether the differences in *k*_cat_/*K*_m_ measured using model substrates could be predictive of the pattern of cleavage of more complex RNAs. Knockout of Nsp15 results in the accumulation of polyU RNAs (PUN) in mutant infected cells, suggesting PUN RNAs produced as negative strand intermediates during replication are substrates (5). PUN_ss28 is a representative PUN substrate that was previously characterized and serves as a reference for comparison of how the local sequence and structure environment can alter site specificity (Table 1) (23,25). Previous analyses of PUN_ss28 cleavage kinetics under multiple turnover conditions (500 nM S, 50 nM Nsp15 pH 7, 150 mM NaCl and 5 mM MnCl_2_) showed a clear preference for UU cleavage (23). To extend these results, we examined the kinetics of PUN_ss28 cleavage at early time points to better resolve the relative initial rates of cleavage at individual sites (Figure 7).

The PUN_ss28 RNA sequence has a 5′ leader of 10 uridine residues, followed by an 18-nucleotide sequence containing potential UG, UA and UU sites, as well as two UC sites (Table 1). The kinetics of PUN_ss28 cleavage are rapid, which is likely due to the presence of 15 potential cleavage sites. Substrate depletion occurs with an observed rate reflecting an apparent *k*_cat_/*K*_m_ of 0.34 × 10^6^ M^−1^s^−1^ calculated by quantifying the initial rate for the decrease in full-length substrate (Table 2). Notably, normalizing this observed *k*_cat_/*K*_m_ to the number of possible U cleavage sites (0.34 × 10^6^ M^−1^s^−1^ / 15 sites) gives a value of 0.022 × 10^6^ M^−1^s^−1^, which is similar to the *k*_cat_/*K*_m_ observed for single site ssRNA substrates (Table 2). The presence of 15 times the number of potential U cleavage sites results in a *k*_cat_/*K*_m_ for PUN_ss28 based on single cleavage kinetics that is only ∼4-fold greater than 1S_bulge, which contains only a single U (0.34 × 10^6^ versus 0.079 × 10^6^ M^−1^s^−1^).

Given the higher *k*_cat_/*K*_m_ for UG and UA cleavage in ssRNA, the expected pattern of initial cleavage of PUN_ss28 should primarily reflect hydrolysis at the two UR dinucleotides. U_10_G and U_21_A cleavage would result in two 5′-Fl labeled fragments (a21 and c10 in Figure 7) and the corresponding 3′-Cy5 labeled products (7a and 18c). However, a distribution of products is observed, representing cleavage in the 5′ poly U leader and a smaller number of products resulting from cleavage elsewhere in the RNA substrate. As the reaction continues, products migrating at positions consistent with internal U_12_C cleavage accumulate, followed by the progressive formation of U_18_C and C_19_C cleavage products, followed by U_15_U and U_16_C.

A significant difference in the kinetics of Nsp15 cleavage at the two UR sites located at U_10_G and U_21_A is readily apparent from the accumulation of 3′-Cy5 labeled cleavage fragments (7a and 18c). Cleavage at U_10_G occurs rapidly, resulting in a 3′ Cy5 labeled product (labeled 18c in Figure 5) that accumulates at early time points. The 18c product contains the U_21_A cleavage site; however, Nsp15 cleavage to generate the 7a product characteristic of U_21_A hydrolysis occurs with slower kinetics. This result contrasts with the relative rates of UG and UA cleavage observed for the 2S_ss20UG/UA substrate, where products from both sites are formed with very similar kinetics. Thus, the polyU and B_+1_ specificities can account for the pattern of initial Nsp15 cleavage of the PUN_ss28 substrate; however, significant deviations, apparently due to the RNA local context, can override these factors.

### Comparative analysis of divalent ion activation of Nsp15 for alternative RNA substrates

**Figure 8.**
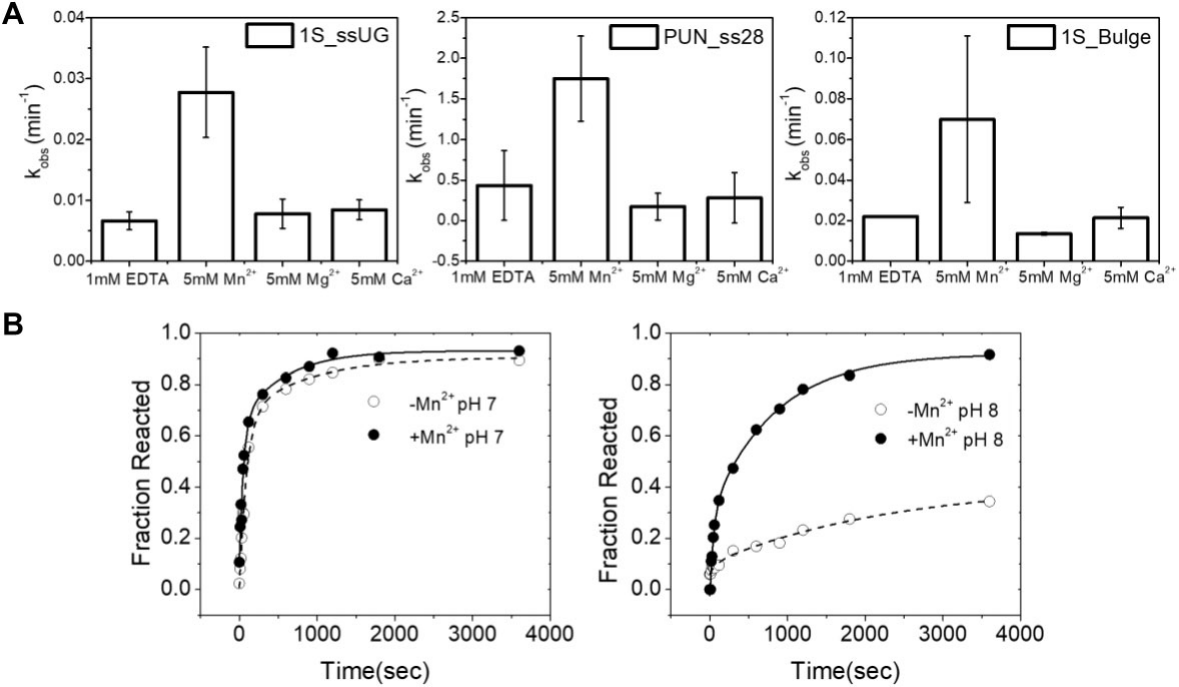
Comparison of divalent metal ion activation of 1S_ssUG, 1S_bulge and PUN_ss28 substrates. The reactions were run under multiple turnover conditions with 0.5 μM RNA substrate, 50 nM Nsp15, and standard reaction buffer containing 5 mM Mn^2+^, Mg^2+^, Ca^2+^ or 1 mM EDTA. (**A**) Bar graph representing the *v*_obs_ versus metal ion for the 1S_ssUG, 1S_bulge and PUN_ss28 RNA substrates, as indicated in the upper right corner of the graph. The observed activation in the presence of Mn^2+^ compared to control reactions containing 1 mM EDTA is approximately 3-fold for the 1S_ssUG substrate, whereas for the PUN_ss28 and 1S_bulge substrate, it is 1.8-fold. Mg^2+^ and Ca^2+^ had minimum or no activation effect for all the substrates compared to reactions containing 1 mM EDTA. (**B**) Plots of 1S_ssUG single turnover kinetics in reactions containing 2 mM Mn^2+^(filled circles) or 1 mM EDTA (open circles) at pH 7 (left) and pH 8 (right). The data for the reaction containing 1 mM EDTA at pH 8.0 is fit to a single exponential (equation 2) while the data for the 1 mM EDTA containing reaction at pH 7.0 were fit to a double exponential (equation 3) (dashed lines). The data for reactions containing 2 mM Mn^2+^ were fit to a double exponential (equation 3) (solid lines).

A characteristic feature of EndoU endonucleases, including Nsp15, is the activation of RNA cleavage by Mn^2+^ (14,26). However, the mechanism of activation and the biological role with respect to Nsp15 function in viral replication are not known. The ability to compare *k*_cat_/*K*_m_ values for alternative model RNA substrates allowed us to quantitatively compare the effect of divalent ions on Nsp15 substrate specificity. Additionally, a comparison of the effects of divalent ions on the multiple turnover rates provides a measure of the potential regulation due to changes in divalent ion type or concentration that may contribute to the viral lifecycle. Accordingly, we determined the extent of divalent metal ion activation for the 1S_ssUG, 1S_bulge and PUN_ss28 substrates under substrate limiting conditions (Figure 8). Under *in vitro* reaction conditions that are near physiological pH and monovalent ion concentration, there is relatively little effect of 5 mM Mg^2+^ or Ca^2+^ on any of the substrates. However, all three RNA substrates showed an increase in the observed rate when 5 mM Mn^2+^ was included in the reaction. Interestingly, the magnitude of divalent ion activation is relatively small, resulting in only a ∼2-fold increase in the multiple turnover rate with no apparent difference between the three substrates.

This result contrasts with previous studies documenting larger increases in Nsp15 activity in the presence of millimolar concentrations of Mn^2+^ (14,26) However, we recently provided kinetic evidence for a model of Mn^2+^ activation, involving stabilization of a more active enzyme conformation and not a direct effect of divalent ions on the rate constant for catalysis, *k*_c_ (25). In this model, divalent ions shift the ensemble of conformations toward the more active state(s). At pH 8, where catalysis is slow insuring that the cleavage step is rate limiting, this results is characteristic biphasic single turnover kinetics representing the rate constants for the fast and slow reacting enzyme conformations.

We hypothesized that the small effect of divalent ion activation on *k*_cat_/*K*_m_ at pH 7 could be due to pH dependent population of the activated enzyme conformation relative to pH 8. In this model, the single turnover kinetics measured at pH 7 should reflect a high fraction of fast reacting Nsp15 in the absence of divalent ions, therefore allowing only a small possible change in the overall distribution toward the active conformation. Accordingly, we compared the effects of 5 mM Mn^2+^ on the single turnover kinetics of 1S_ssUG RNA at pH 8 and pH 7 to test for differences in the fast and slow reacting conformations assayed by biphasic kinetics (Figure 8). As previously observed, the reaction kinetics at pH 8.0 in the presence of EDTA are monophasic and fit a single exponential while adding Mn^2+^ results in biphasic kinetics. As shown in Supplementary Figure S3 the presence of 2 mM Mn^2+^ results in biphasic kinetics as evaluated by comparing the fitting to both single and double exponential equations. The *k*_c_ from fitting to a single exponential equation (equation 2) or the rate constants for the fast (*k*_1_) and slow (*k*_2_) phases determined by fitting to a double exponential function (equation 3) are compared in Supplementary Table S3. Consistent with previous results, at pH 8.0 the addition of Mn^2+^ results in a fast-reacting phase (*k*_1_ 0.011 ± 0.002 s^−1^) and a smaller phase with slower kinetics rate constant similar to *k*_c_ measured in EDTA (*k*_1_ 0.0012 ± 0.0001 s^−1^ versus *k*_c_ 0.0006 ± 0.0001 s^−1^). However, at pH 7.0, we observe essentially identical single turnover kinetics in the presence and absence of Mn^2+^ and similar rate constants for the fast and slow phases (Supplementary Table S3). Thus, under these conditions, the addition of divalent ions does not appear to alter either the reaction rate constants or the distribution of ES complexes in the fast and slow phases.

These results indicate that lowering the pH increases the ensemble of enzymes that react with fast kinetics in the absence of divalent ions. Therefore, further activation by Mn^2+^ is therefore incremental because most of the enzyme already exists in the activated form. This model is generally consistent with reports of the pH dependent formation of an open and relatively static conformation of Nsp15 at pH 7.5 observed by x-ray crystallography (18). Independent of the underlying mechanism, it is important to note that under the conditions tested here, there is only a ∼2-fold increase in multiple turnover cleavage in the presence of Mn^2+^, despite significant differences in the RNA structure of the three alternative substrates tested.

## Discussion

There have been significant advances in understanding Nsp15 structure (2,29,31), its role in host immune evasion (6,49) and discovery of inhibitors to combat viral pathogenesis (18,50,51). Yet, its kinetics and the principles of specificity that determine how it identifies *in vivo* targets are just beginning to become clear. Several recent studies have demonstrated an important role for RNA structure in directing Nsp15 to alternative cleavage sites (28,29,31). These results are foundational in revealing a preference for bulged U residues and they further demonstrate large differences in the rates of cleavage at alternative sites due to differences in surrounding RNA structure. Here, we further advance our understanding of the role of RNA sequence and structure in directing Nsp15 specificity by systematically comparing the *k*_cat_/*K*_m_ values for a series of model RNA substrates. Each model RNA substrate was designed to interrogate specific features of Nsp15 specificity, including U positioning within RNA helical structure and location within linear ssRNA. These new data unambiguously demonstrate that Nsp15 functions primarily as an RNA structure-specific ribonuclease and provide an initial set of benchmark values for the contribution of sequence and structure. The results show that the nucleobase located 3′ to the cleavage site can dictate cleavage site specificity within ssRNA substrates. Our results confirm that pairing adjacent to a bulged U acts as a positive determinant and further show that it increases *k*_cat_/*K*_m_ relative to a U within a ssRNA context by nearly an order of magnitude. However, U pairing within dsRNA structure acts as a potent anti-determinant that can essentially block cleavage at an otherwise reactive U sequence. Remarkably, despite large differences in *k*_cat_/*K*_m_ for alternative substrates the cleavage step is largely insensitive to RNA structure. This result demonstrates that the chemical step is not rate limiting for multiple turnover reactions, and that Nsp15 specificity is due to differences in the kinetics of substrate association or a conformational change. By analyzing the kinetics of multisite substrates, we further demonstrate that the binding site for ssRNA extends 3–4 nucleotides on either side of the cleavage site and provide evidence that cleavage at otherwise optimal cleavage site sequences can be suppressed by more numerous polyU sequences within the same RNA. The preference for structured RNA and competition between alternative sites demonstrated here will necessarily define to a large degree how Nsp15 recognizes the appropriate cleavage sites within the viral RNA genome. In addition, the kinetic and mechanistic insights gained provide a framework for defining the RNA-protein interactions that enforce substrate specificity and can allow more detailed understanding of mechanisms of inhibition.

Previous studies have investigated how sequences flanking the cleavage site U can affect the rate of Nsp15 cleavage and order of site selection within multi-site substrate RNAs, and that structure can also dictate cleavage site specificity (23,28–30). Our results place quantitative limits on B_+1_ specificity, U position within ssRNA, and dsRNA structure that are fully consistent with these previous studies. A comparison of multiple turnover kinetics shows that B_+1_ specificity accounts for a several fold contribution to *k*_cat_/*K*_m_, with a preference for UA and UG consistent with previously established rules of Nsp15 specificity. Additionally, we detect little apparent contribution from the location of U sites along the length of an unstructured RNA. In contrast to the ∼3 fold effect of B_+1_ specificity, helical structure can contribute orders of magnitude to *k*_cat_/*K*_m_ relative to a ssRNA context and can act as a dominant anti-determinant to sequester and suppress cleavage at an otherwise reactive U residue. Thus, in terms of evaluating the magnitude of the energetics and mechanistic contributions of RNA structure to Nsp15 reaction kinetics the presence of only two basepairs on the other site of a bulged U is sufficient to impart an order of magnitude rate enhancement. This result is consistent with a recent structure model of Nsp15 bound to a 52-mer dsRNA substrate which shows contacts between two monomers and 2–3 basepairs on either side of the bulged U in the active site (29). The ability to engage additional contacts thus provides a rational for the faster reaction kinetics. The fact that RNA-protein contacts more distal to the cleavage site are also observed suggests Nsp15 may be optimized for larger more complex substrates.

The relatively large range of effects that local RNA structure can have on cleavage rate even for unpaired U residues is recently demonstrated by results comparing the effects of target U location within the GNRNA pentaloop of a model viral RNA (28). A U at the center nucleotide is most reactive while a U adjacent to the paired stem was shown to be largely refractory to cleavage. These results support the general model in which U residues within structurally constrained geometries are less able to engage with the Nsp15 active site. Consistent with the generally negative effects of restricting conformational flexibility, we observe that 1S_loop (which forms a compact GUAA tetraloop) is processed with a *k*_cat_/*K*_m_ that is at least an order of magnitude less than 1S_ssUG. The greater *k*_cat_/*K*_m_ for 1S_bulge relative to 1S_ssUG (0.079 ± 0.002 × 10^6^ M^−1^s^−1^ versus 0.0090 ± 0.001 × 10^6^ M^−1^s^−1^) provides quantitative evidence consistent with the proposal that a structural context facilitates optimal positioning of the scissile nucleotide in the catalytic pocket of Nsp15. Notably, the *k*_cat_/*K*_m_ for cleavage of a minimal UpG dinucleotide (0.015 × 10^6^ M^−1^s^−1^ ) is similar to the value for the 1S_ssUG oligonucleotide(25), and equivalent rates of cleavage are observed for each internal U with the 6S_ss22 RNA. Together these data underscore that in the absence of higher order structure, the contribution of flanking sequences beyond the 3′ B_+1_ nucleotide is minimal. Thus, understanding Nsp15 specificity will at some level require accounting for a spectrum of different *k*_cat_/*K*_m_ values depending on sequence and structure, as well as the population of alternative conformations.

Activation of Nsp15 activity by Mn^2+^ is well documented and divalent metal ion activation is a defining characteristic of the class of EndoU endonuclease to which it belongs. (14,26) However, a mechanistic understanding of how divalent ions enhance Nsp15 endonuclease activity is lacking. To date, binding sites for divalent ions have yet to be observed in structures of Nsp15 alone or in complex with RNA. Additionally, metal ions are dispensable for *in vitro* activity and both structural and biochemical evidence demonstrate that the active site employs a metal ion independent acid/base catalytic mechanism (25). While there is evidence for conformational dynamics in the free enzyme that involves the catalytic domain *e.g*.(20,45), the potential for metal ion induced changes in structure and dynamics is largely unexplored. Using the framework provided by our comparison of the *k*_cat_/*K*_m_ values for alternative model RNA substrates we measured the level of divalent ion activation and asked whether activation might depend on RNA structure. Surprisingly, we report that Mn^2+^ activation of Nsp15 is only ∼2-fold on multiple turnover kinetics for all three of the substrates tested. Moreover, no detectable activation was observed using either Mg^2+^ or Ca^2+^. A general model of Mn^2+^ activation consistent with single turnover kinetics results involves stabilization of a more active enzyme conformation rather than a direct effect on catalysis (25). A change in biphasic kinetics in the presence of Mn^2+^ provided evidence for a shift in the ensemble of conformations toward the more active state. Here, we provide additional kinetic evidence that protonation of one or more enzyme functional groups can also result in a similar change in the distribution of the enzyme population. The net result is that under physiological conditions divalent ions do not appear to alter the reaction rate constants or the distribution of ES complexes in the fast and slow phases. Interestingly, pH dependent formation of an open and relatively static conformation of Nsp15 at pH 7.5 has been observed by X-ray crystallography (18). Together, these data suggest there may be multiple routes to Nsp15 activation that are linked to small ion binding or protonation. As these effects appear to be linked to protein conformational changes, raising the intriguing possibility that binding of proteins, RNAs, or other ligands might act allosterically to induce alternative Nsp15 conformations with different activities.

Together with previous results, the characterization of reaction kinetics for alternative substrates provides a key step toward identifying and understanding the function of physiological substrates of Nsp15. A key question considering recent results is, where would Nsp15 encounter bulged uridines during viral replication within a host? One possibility is the presence of bulged uridines within the viral (+) RNA genome. Another possibility lies in the polyA/U sequence interaction that occurs during transcription of the viral genome. Uridine and adenosine residues create weak interactions together and thus it would be easier for a uridine to flip-out of this interaction or create bulged uridine residues. Finally, the polyU leader sequences on the 5′ end of the (-) gRNA has been shown to form dsRNA structures with AG rich sequences. It is highly likely that bulged uridines result from this folding process. Any of these three could be potential physiological substrates for Nsp15 and align with the role of Nsp15 to limit the activation of a host immune response by regulating the accumulation of dsRNA during viral replication. Nsp15 cleavage sites have previously been mapped in the MHV genome following viral infection(22). Only a minor subset of potential sites in the vast background of competing potential sites were detected consistent with specific recognition of sites matching a consensus motif. As yet, RNA structure prediction and motif analyses have not identified conserved structural elements at nsp15 cleavage sites or within flanking sequences (28). In general, segments of genomic RNA with lower predicted thermodynamic stability are more susceptible to Nsp15 cleavage, and quantitative studies remain primarily confined to minimal model RNAs. Importantly, for both *in vivo* and *in vitro* mapping of Nsp15 cleavage sites the ensemble nature of RNA structure is likely to confound a straightforward interpretation.

Given the extreme sensitivity to RNA structure and dynamics of RNA folding, it follows that even transiently forming dsRNA structure could give rise to differences in cleavage rates between apparently otherwise ssRNA substrates and sites within substrates. It is well established that RNA molecules can undergo conformational changes in response to various environmental stimuli, including temperature, pH, ligand binding and protein binding. Such differences in the induced conformations can have orders of magnitude effects on Nsp15 recognition of individual U cleavage sites. Divalent ions and many other potential cellular signals that affect the transcriptome and viral RNA could therefore alter the pattern of Nsp15 cleavage. Moreover, the ratio of reaction rates for alternative substrates is proportional to the ratio of the substrate concentrations and their *k_cat_*/*K_m_* values. It therefore follows that the presence of high concentrations of substrates with relatively low *k_cat_*/*K_m_* can effectively suppress cleavage at otherwise reactive sites with higher *k_cat_*/*K_m_*. The effect of a higher effective concentration of target sites with lower *k_cat_*/*K_m_* is consistent with the observed kinetics of the PUN substrate in which the polyU leader mimicking the viral negative strand is preferentially cleaved over the intrinsically more reactive UA/G sequences. Thus, the very large difference in *k*_cat_/*K*_m_ between a dsRNA, ssRNA and bulged RNA substrates that we report show that Nsp15 specificity is more complex than originally believed and is likely to be regulated by factors that alter RNA conformation and that affects competition between alternative substrates.

Thus, the systematic kinetic analyses of model substrates described here advances our understanding of the role of RNA sequence and structure in directing Nsp15 specificity in several key ways. These new data confirm and advance previous studies of Nsp15 specificity and further provide quantitative limits on the contributions of sequence, structure and cleavage site location essential for defining optimal substrates and developing quantitative models of specificity. The kinetic and mechanistic insights provide a context for structure-function studies aimed at defining the RNA-protein interactions that underlie specificity, determining how alternative substrates are distinguished and interrogating mechanisms of inhibition. Investigation of Nsp15 single turnover kinetics provides support for multiple routes to Nsp15 activation that are linked to small ion binding or protonation and raises questions regarding the potential importance of divalent ion activation *in vivo*. The most important implication emerging from these and other studies aimed at defining the substrate specificity of Nsp15 is that any accurate model of substrate selection must necessarily account for the ensemble nature of RNA structure.

## Supplementary Material

gkae939_Supplemental_File

## Data Availability

All data produced in this study are fully available without restriction.
