# Supplementary material for: Alternative substrate kinetics of SARS-CoV-2 Nsp15 endonuclease reveals a specificity landscape dominated by RNA structure
Source: Nucleic Acids Res. 2024 Oct 30;52(21):13419–33. doi: 10.1093/nar/gkae939 (PMC11602132; doi:10.1093/nar/gkae939)
Supplement: gkae939_Supplemental_File [file gkae939_supplemental_file.pdf]

SUPPLEMENTARY INFORMATION

Alternative Substrate Kinetics of SARS-CoV-2 Nsp15 Endonuclease Reveals a Specificity Landscape Dominated by RNA Structure

Nidhi Kalia, Kimberly C Snell, Michael E Harris

Department of Chemistry, University of Florida, Gainesville, FL 32611

Contents

Figure S1. Determination of Nsp15 reaction kinetics from fluorescence data. 2

Table S1. Observed v_{ss} (min^{-1}) for model RNA substrates at 0.25 – 30 μM determined by analysis of fluorescence data or gel data for 1S_bulge. 3

Figure S2. Determination of k_{cat}/K_m for 1S_ssUG, 1S_ssUA, 1S_ssUC and 2S_ss21UG/UC using the Michaelis-Menten equation. 4

Table S2. Comparison of k_{cat}/K_m values determined by fitting to a linear equation versus the Michaelis-Menten (MM) equation. 5

Figure S3. Comparison of fitting 1S_ssUG single turnover data in EDTA and Mn^{2+} to single and double exponential functions. 6

Table S3. Observed rate constants from single or double exponential fitting of 1S_ssUG single turnover kinetics. 7

Figure S1. Determination of Nsp15 reaction kinetics from fluorescence data.

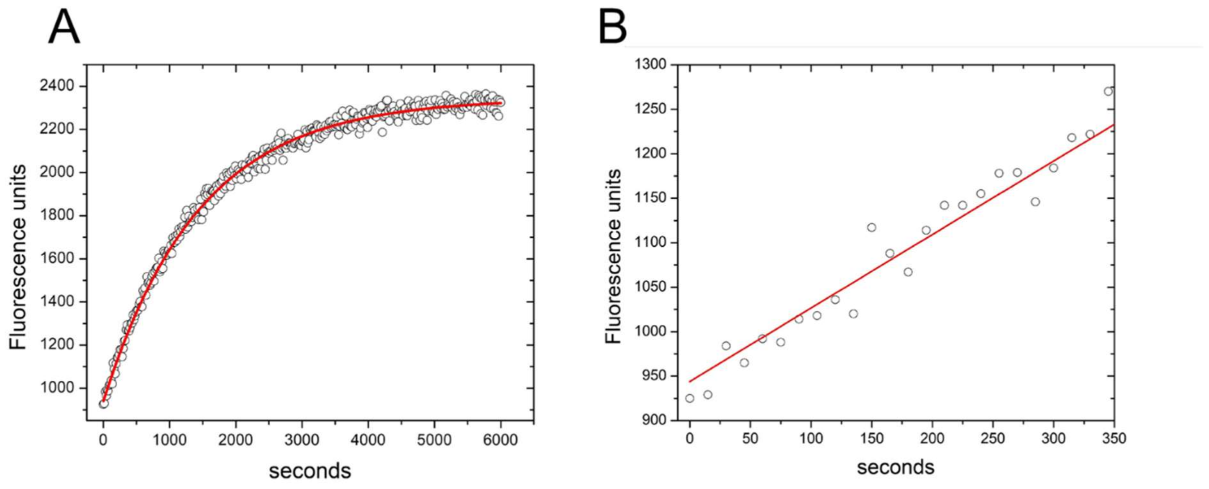

Figure S1. Determination of reaction kinetics from fluorescence data. (**A**) Example of fluorescence emission signal data obtained for Nsp15 cleavage of model substrate RNA. The reaction shown is for the multiple turnover reaction of 500 nM 1S_ssUG_PL RNA with fluorescence measured by plate reader as described in Materials and Methods. The red line shows a fit of the data to a single exponential function (equation 2) with a rate constant of 0.042 min^{-1} (R^2 0.99). (**B**) Detail showing the first 10% of the observed reaction amplitude. The red line shows a fit of the data to a linear function (R^2 0.97) reflecting an initial rate of 0.035 min^{-1}.

Table S1. Observed v_{ss} (min^{-1}) for model RNA substrates at 0.25 – 30 μM determined by analysis of fluorescence data or gel data for 1S_bulge.

	V_{ss} min^{-1}					
**[S]**	1S_bulge	1S_ssUG	1S_ssUA	1S_ssUC	1S_ssUG/UC	PUN_ss28
0.25 μM	4.4 ± 1.5	0.208 ± 0.002	0.212 ± 0.002	0.124 ± 0.002	0.78 ± 0.05	3.9 ± 0.1
0.5 μM	2.1 ± 0.3	0.448 ± 0.002	0.48 ± 0.01	0.205 ± 0.003	1.3 ± 0.1	5.28 ± 0.08
1 μM	3.1 ± 1.2	1.20 ± 0.01	1.32 ± 0.01	0.45 ± 0.01	2.5 ± 0.1	11.0 ± 0.4
2 μM	5.0 ± 1.5	1.64 ± 0.03	1.95 ± 0.03	0.90 ± 0.01	4.2 ± 0.2	19.2 ± 0.2
5 μM	29 ± 6	3.43 ± 0.01	2.39 ± 0.02	1.89 ± 0.03	12.2 ± 1.4	108 ± 5
10 μM	51 ± 5	6.58 ± 0.01	3.73 ± 0.04	2.29 ± 0.02		156 ± 6
20 μM		11.0 ± 0.1	4.63 ± 0.08	4.99 ± 0.04	24 ± 3.2	420 ± 24
30 μM	143 ± 11					

Figure S2. Determination of k_{cat}/K_m for 1S_ssUG, 1S_ssUA, 1S_ssUC and 2S_ss21UG/UC using the Michaelis-Menten equation.

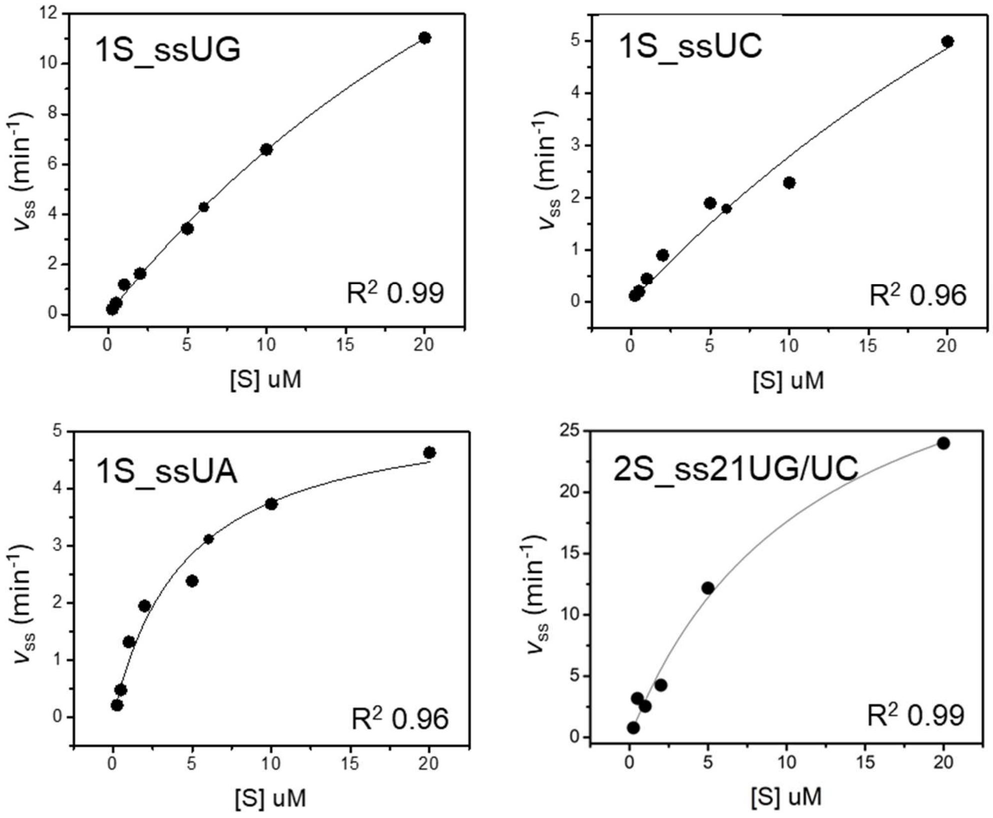

Figure S2. Determination of k_{cat}/K_m for 1S_ssUG, 1S_ssUA, 1S_ssUC, and 2S_ss21UG/UC using the Michaelis-Menten equation. The initial multiple turnover rates determined using the fluorescence-based plate reader assay from **Table S1** were fit to equation 1 to evaluate k_{cat} and K_m. The standard errors are reported in **Table S2**.

Table S2. Comparison of k_{cat}/K_m values determined by fitting to a linear equation versus the Michaelis-Menten (MM) equation.

Substrate	k_{cat}/K_m (MM) x 10^6 $M^{-1}s^{-1}$	k_{cat}/K_m (linear) x 10^6 $M^{-1}s^{-1}$
**1S_bulge**	n.a.	0.079 ± 0.002
**1S_ssUG**	0.013 ± 0.003	0.009 ± 0.0003
**1S_ssUA**	0.002 ± 0.001	0.016 ± 0.003
**1S_ssUC**	0.006 ± 0.006	0.0039 ± 0.0003
**1S_ssUG/UC**	0.052 ± 0.005	0.017 ± 0.004
**PUN_ss28**	n.a.	0.34 ± 0.02

Figure S3. Comparison of fitting 1S_ssUG single turnover data in EDTA and Mn^{2+} to single and double exponential functions.

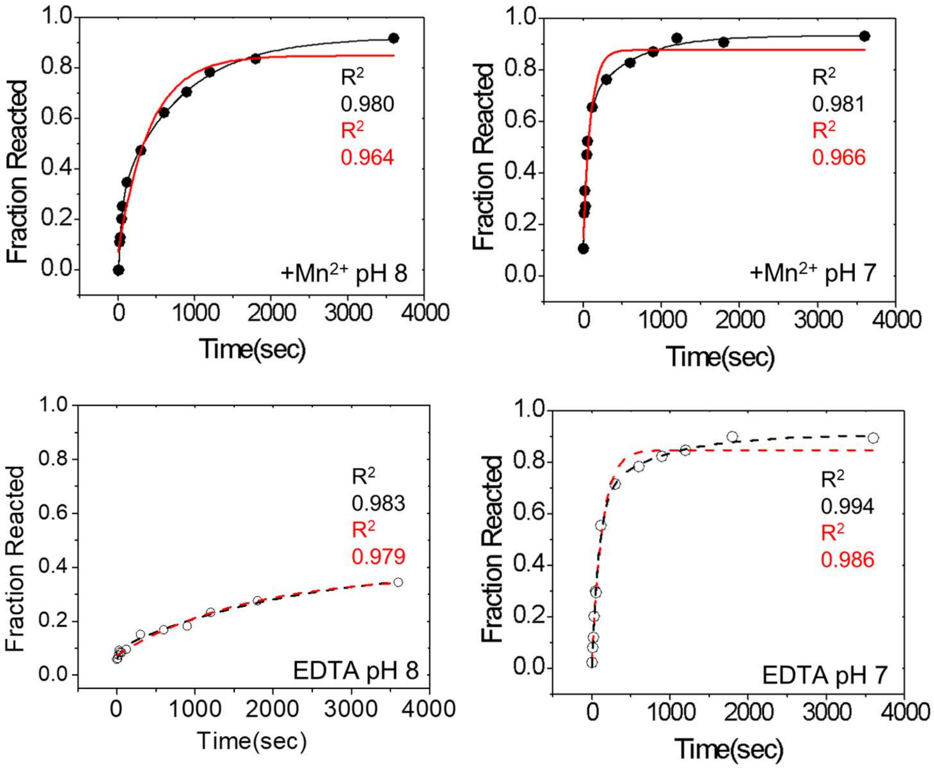

Figure S3. Comparison of fitting 1S_ssUG single turnover data in EDTA and Mn^{2+} to single and double exponential functions. Single turnover data shown in **Figure 8** fit for reactions in 2 mM Mn^{2+} (filled symbols) or 1 mM EDTA (open symbols) are shown. The results of fitting data to a single exponential function are shown in red and results for fitting to a double exponential function are in black. The R^2 values for fitting either equation are shown in the matching color. The k_{obs} from the single exponential function fitting and the k_1 and k_2 for the fast and slow phases, respectively, are shown in **Table S3**.

Table S3. Observed rate constants from single or double exponential fitting of 1S_ssUG single turnover kinetics.

	SINGLE EXP FIT	DOUBLE EXP FIT	
Reaction	k_{obs} s^{-1}	k_1 s^{-1}	k_2 s^{-1}
2 mM Mn^{2+}; pH 8	0.0024 ± 0.0004	0.011 ± 0.002	0.0012 ± 0.0001
1 mM EDTA; pH 8	0.0006 ± 0.0001	0.012 ± 0.011	0.00043 ± 0.00015
2 mM Mn^{2+}; pH 7	0.008 ± 0.001	0.020 ± 0.004	0.0012 ± 0.0007
1 mM EDTA; pH 7	0.010 ± 0.002	0.019 ± 0.006	0.0017 ± 0.0001
